# Expectations of breast-conserving therapy: a qualitative study

**DOI:** 10.1186/s41687-019-0167-5

**Published:** 2019-12-27

**Authors:** Sarah Fuzesi, Karima Becetti, Anne F. Klassen, Mary L. Gemignani, Andrea L. Pusic

**Affiliations:** 1 Department of Surgery, Memorial Sloan Kettering Cancer Center, 1275 York Avenue, New York, NY 10065 USA; 2 Department of Surgery, Saint Barnabas Medical Center, 94 Old Short Hills Road, Livingston, NJ 07039 USA; 3 Hospital for Special Surgery, 535 East 70th Street, New York, NY 10021 USA; 4 Department of Pediatrics, McMaster University, 1280 Main Street W, Hamilton, ON L8S 4L8 Canada; 5 Dana-Farber/Brigham and Women’s Cancer Center, Boston, MA 02115 USA

**Keywords:** Breast cancer, Breast-conserving therapy, Health-related quality of life, Expectations, Patient-reported measure, Preoperative education

## Abstract

**Background:**

Early-stage breast cancer is often treated with breast-conserving therapy (BCT), including lumpectomy with radiation therapy. Patients’ expectations of BCT remain largely unknown. Expectations affect perceptions of treatment-related experiences and health-related quality of life (HR-QOL) outcomes. Our primary aim was to describe expectations of BCT among patients with early breast cancer through qualitative methods. Our secondary aim was to inform preoperative patient education and improve the patient experience through knowledge.

**Methods:**

We used a grounded-theory approach to investigate a convenience sample of 22 women with stage I and II breast cancer who were treated with BCT at a single hospital in New York City between May and August 2016. Semi-structured interviews were conducted in person and by telephone. Open-ended questions covered participants’ expectations of treatment experiences and outcomes. Data was analyzed in a line-by-line approach to identify emergent themes related to patient expectations. Interviews continued until no new themes emerged.

**Results:**

Analysis of data identified the following themes related to patient expectations of BCT: experience of cancer care, recovery, appearance, and HR-QOL. Despite preoperative informed consent and teaching, participants expressed few expectations preoperatively, owing to a lack of knowledge about the process of care. Lack of expectations preoperatively was compensated with available care and resources postoperatively.

**Conclusions:**

Patients in our sample had a surprisingly limited understanding of what to expect during treatment with BCT. Despite available information and preoperative teaching, patients have a clear knowledge gap regarding BCT. These findings suggest patients often undergo cancer treatment with trust rather than complete understanding of the process. This data may be used to enhance preoperative discussions aimed at preparing patients for surgery and treatment.

## Background

Breast cancer is the second most common cancer among women in the United States [27]. Early-stage breast cancer is often treated with breast-conserving therapy (BCT), including lumpectomy with radiation therapy or mastectomy with or without breast reconstruction. Despite high survival rates and improved cosmetic outcomes, treatment for breast cancer exerts a psychological and physical impact on women [4, 5, 13, 17]. As a result, discussions about expectations regarding health-related quality of life (HR-QOL) outcomes are an increasingly important part of preoperative education and shared decision-making [19, 26].

Patient satisfaction with treatment outcomes and long-term HR-QOL vary by many factors, including age, race, ethnicity, education, and preoperative counseling [6, 19]. Expectations have been shown to affect postoperative satisfaction and HR-QOL outcomes in other surgical groups [7, 29]; however the research on breast surgery is limited [7, 23–25, 29, 30]. Researchers have found that most patients undergoing mastectomy with breast reconstruction did not know what to expect or had expectations that were discordant from those of clinicians, despite preoperative teaching [7, 24]. Lee et al. found that women undergoing surgery for breast cancer lacked knowledge about local recurrence, survival, and re-excision [15]. BCT may have fewer psychological consequences than mastectomy [1–3]. Waljee et al. found that patients who underwent BCT underestimated HR-QOL and overestimated stigma following BCT [30]. Teaching tools may help align expectations of HR-QOL with outcomes and improve the patient experience overall.

The purpose of this study was to explore HR-QOL expectations of patients with early breast cancer undergoing BCT including surgery and radiation. Clinicians aiming to improve satisfaction with overall care will benefit from better understanding what patients expect from treatment as well as what they feel is important to their care. Content generated from patients’ own experiences can enrich preoperative education and help prepare future patients undergoing BCT.

## Patients and methods

### Study design and participants

Ethics approval was obtained from the Memorial Sloan Kettering Institutional Review Board before initiation of the study. Potential participants were approached by the sole interviewer (S.F.) during a regularly scheduled appointment and were invited to participate. Women were eligible if they had early-stage breast cancer, were planning to have BCT or had undergone BCT within 6–12 months, spoke English, were able to participate in an interview, and were aged 18–75 years. There were two groups of patients. Participants who were interviewed at their preoperative surgical visit and were invited to participate in four interviews total, (before surgery, 6 weeks, 3 months, and 6 months after surgery). Having participants interviewed multiple times allowed us to explore unexpected experiences at different stages of treatment and recovery. The second group were participants who had already undergone treatment and were interviewed once, at a follow-up appointment.

### Interview

We used a grounded-theory approach. An interview guide was developed on the basis of previous work [7, 12, 22–24]. After informed consent was obtained, participants were interviewed by an experienced qualitative researcher. Participants were asked open-ended questions about their decision to have surgery as well as their expectations of lumpectomy and radiation. Specific questions focused on expectations of breast appearance, recovery period, psychological and social impact, and sexual well-being. These themes were the same for interviews conducted postoperatively, however, participants were asked to recall their expectations and whether their experiences differed from their expectations. Interviews were conducted either at the hospital, in a one-to-one fashion, or at home, over the telephone. Interviews were digitally recorded and transcribed verbatim, with all identifiable information excluded.

### Data collection and analysis

Data collection and analysis took place concurrently so that findings from earlier interviews could inform subsequent data collection. Data were analyzed for emergent themes relating to expectations of BCT, and a coding structure was developed. During the first step of analysis, also called “open coding,” two trained researchers independently coded the data line by line. Interview transcripts were analyzed in sets of five during meetings of the research team. Constant comparison was performed to ensure codes and categories for emergent themes were consistent between the two researchers. Input on the codebook was obtained from the research team. This process of coding continued with new transcripts, and relationships between codes and categories were further refined. Interviews continued until no new themes emerged. NVivo 11 software (QSR International, Melbourne, Australia) was used for data management.

## Results

### Demographics

Twenty-seven people were approached for this study and 22 participants agreed to participate. The sample included 9 participants recruited before BCT (interviewed before surgery and up to three times after surgery) and 13 participants recruited after BCT (interviewed once) (Table 1). In total, 30 interviews were performed. Of the participants interviewed before surgery, one did not agree to participate in any further interviews and two were lost to followup. Thirteen participants were interviewed once at a follow up appointment within 1 year of their surgery. We included one participant age 77 because she had undergone lumpectomy with radiation, and we felt her experience was similar to our patient population.

**Table 1:** Participant demographic and clinical characteristics (*n* = 22, mean age = 58)

Characteristic	No. (%)
Age (range, mean)	46–77(57)
Interview timing	
Pre and postoperative	9 (41)
Postoperative only	13 (59)
Stage	
0	3 (14)
I	16 (73)
II	3 (14)
Axillary procedure	
Sentinel lymph node biopsy	19 (86)
None	3 (14)
Radiation therapy	21 (95)
Chemotherapy	7 (32)
Hormone therapy	16 (73)
Marital status	
Married/partner	16 (73)
Divorced/single	5 (23)
Widowed	1 (5)
Race/ethnicity	
White	15 (68)
Black	2 (9)
Hispanic	2 (9)
Other	3 (14)

### Themes

**Fig. 1:**
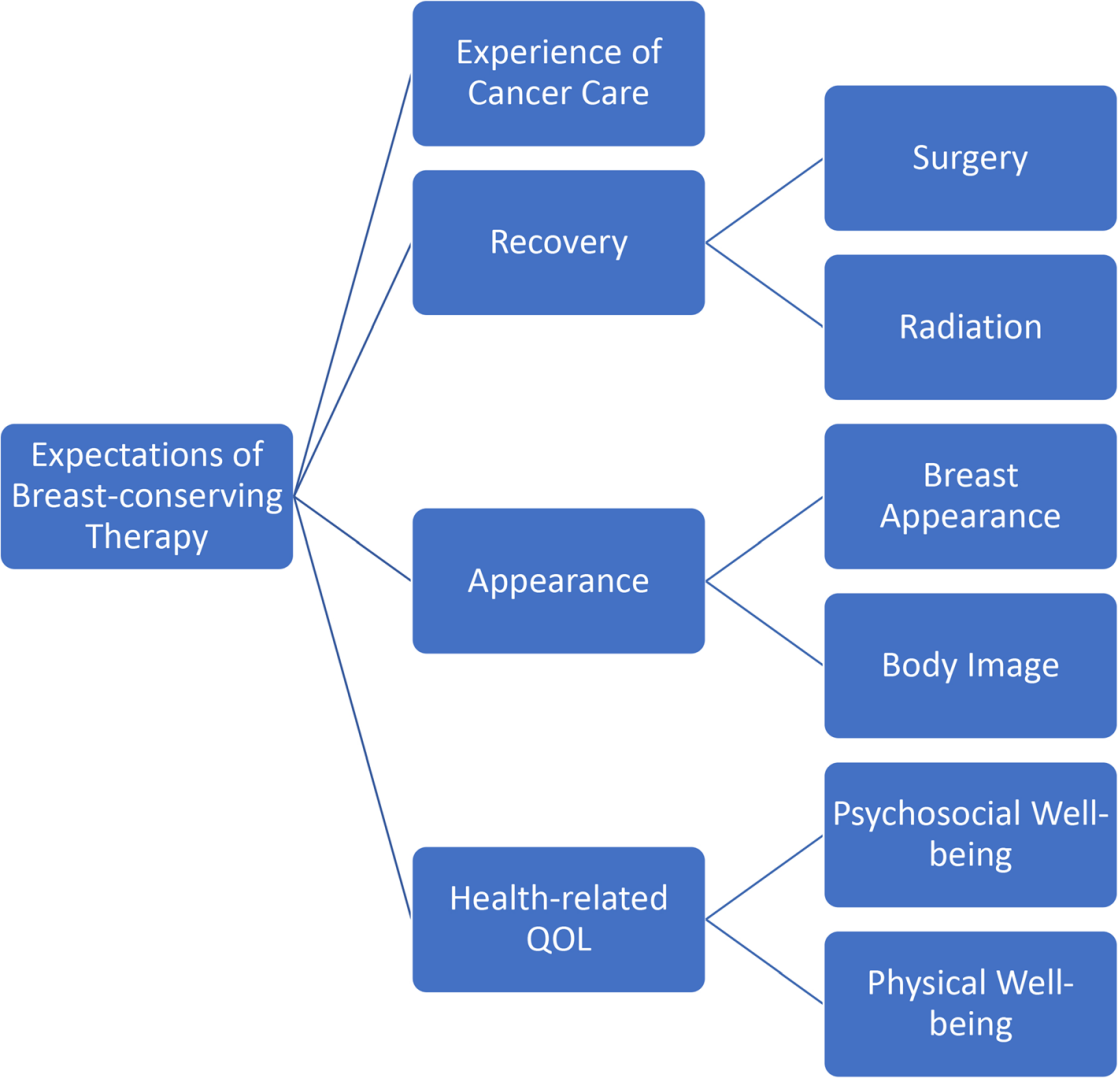
Conceptual Framework

The analysis revealed participant expectations in the following areas: experience of cancer care, recovery, appearance, and HR-QOL. On the basis of these themes, we developed a conceptual framework (Fig. 1).

Preoperative participants had difficulty detailing their expectations (Table 2). These participants experienced high levels of anxiety, and most had difficulty imagining how their life might change beyond the initial postoperative period. They expressed a lack of knowledge about the effects that treatment would have on HR-QOL as well as overall uncertainty about cancer care. Participants who had undergone surgery for other conditions (2 participants) expressed specific expectations about surgical care and recovery. Participants who were still in shock over their cancer diagnosis or who had reported experiencing a high level of anxiety were unable to describe any expectations beyond having the cancer removed.

**Table 2:** Example quotations illustrating participant expectations of BCT

Theme; Subtheme	Example Quotation
Experience of Cancer Care	“So I’m expecting them to say that it’s good to go and hopefully…get the process started with the radiation, and I don’t know how quickly that moves along.” (6 weeks postoperative, Age 65)
Recovery; Surgery	“I was instructed, the more you move, the better—of course, within reason. And that’s what I did. And I had complete range of motion within a couple days.” (13 months postoperative, Age 59)
Recovery; Radiation	“Well, radiation—actually, this was my first time. I had no expectation.” (6 months postoperative, Age 57)
Recovery; Radiation	“I think that it leaves a burn mark. On my sister it was a burn patch. You could see that it was burnt. So I’m expecting that.” (Preoperative, Age 53)
Appearance; Breast Appearance	“I’m not sure what to expect. It didn’t sound too invasive. And the tumor itself is not that big. So I don’t know.” (Preoperative, Age 49).
Appearance; Breast Appearance	“I was concerned, yes. I was concerned about it because I like my [breasts], honest, I do, and that I was going to be slightly disfigured or it was going to be a big difference in size.” (6 months postoperative, Age 46).
HR-QOL; Psychosocial Well-being	“[My] social and work life are very much connected, so I don’t think it impacts anything else, other than the people. They’re a little bit more concerned now than they were before, and the same holds for me.” (Preoperative, Age 49)
HR-QOL; Sexual Well-being	“This is cancer. I’ll deal with that after I get well. [Hormone therapy] gives you menopause. But I was already sort of going through menopause. So…it was the last thing on my mind, actually.” (13 months postoperative, Age 53)

*“I’m not sure I have expectations. I have the hope that it resolves the issue and that, with the radiation follow-up, that that would be it for now.” (Preoperative, Age 49)*

Some participants expressed indifference to concerns about breast appearance (7 participants) and sexual function (8 participants). In contrast, postoperative participants said that BCT had had a profound effect on HR-QOL. Reflecting on their experiences, the postoperative participants described how surgery and radiation affected their lives in ways they didn’t expect, including changes in body image, social confidence, and sexual well-being.

### Experience of cancer care

Preoperative and postoperative participants discussed satisfaction with the experience of cancer care as well as expectations they felt were not met. Preoperatively, participants were focused on their upcoming surgery and expressed expectations about hospital care, including the process of undergoing surgery and the availability of the medical team on the day of surgery (5 participants). A benefit of choosing BCT identified by participants was the ability to have an outpatient procedure and a short recovery period before starting radiation. Participants commented on their expectations of smoothly moving through treatment phases.

*“My expectation [was] that it—the procedure—would have been quick, easy, [and] simple, based on what I understand.” (6 months postoperative, Age 57)*

Postoperatively, participants acknowledged that, although surgery can be quick and easy, each treatment posed another challenge, and they relied on social support and their doctors throughout the process.

Participants expected members of the medical team, including surgeons, radiation oncologists, nurses, and other support staff, to be supportive, informative, and reliable (22 participants). Postoperatively, their expectations were often fulfilled, expressing trust in and great appreciation for their medical team. Participants were unsatisfied with their medical team when they encountered unexpected events, such as unexpected side effects, having trouble understanding recovery instructions (e.g., when to remove bandages), experiencing changes to the treatment regimen, or feeling confused about follow-up appointments and tests (12 participants). Participants identified a learning curve required to navigate the system of multidisciplinary cancer care.

### Recovery

Comments about physical function were mainly related to side effects of treatment to and recovery from surgery or radiation. Preoperatively, participants often did not know what to expect or did not expect great physical changes from surgery or radiation. Few preoperative participants expected to be able to return to their normal routine and activities immediately (2 participants), whereas others had more realistic expectations, such as needing a few days to a week before resuming activities (6 participants).

*“So I don’t know, but I don’t think I will be able to cook much, because it’s my right breast and I’m not lefty, I’m right-handed.” (Preoperative, Age 60)*

*“I was overprepared, because I expected to be in worse shape. So I had family at the house to help me. I didn’t need any help at all. I was able to clean and cook and, you know, do the things that I needed to do right after.” (6 months postoperative, Age 57)*

Participants who had previously experienced surgery in any capacity were more prepared for what to expect in terms of recovery. After completing treatment, participants reflected that they found the surgery to be much easier than other phases of their care, such as radiation, hormone therapy, and chemotherapy.

Participants noted that expectations of pain and physical activity were a main focus of the preoperative teaching they received. Postoperatively, participants expressed satisfaction with and even surprise about the minimal level of pain experienced. Some participants expected pain to be minimal and well-controlled (e.g., not needing to take pain medicine) and did not expect pain to restrict them from activities of daily living and light exercise (8 participants).

*“Probably similar to the sense that you’re uncomfortable. You have pain. That’s the part that’s not bothering me as much…. My body probably doesn’t react with a lot of pain, so I’ll probably be able to deal with it.” (Preoperative, Age 62)*

Some participants expected to experience limitations relating to lifting or using the arm on the side of surgery (4 participants). Three patients with previous injuries that might affect their recovery, such as chronic shoulder or back pain, were the most unsure about recovery and expected recovery to take longer after surgery. Postoperatively, participants were surprised by prolonged breast swelling and numbness, which was often still present 6 months after surgery.

Since the preoperative information and teaching provided by the surgical team focused on recovery from surgery, participants often did not know what to expect from radiation. Some participants expressed specific expectations about side effects of radiation, such as skin irritation and fatigue (7 participants). In general, participants had not yet seen a radiation oncologist, and, therefore, they were unclear about the effects of daily radiation.

*“Every time I ask anybody, everybody’s kind of nonspecific on that, saying that you’ll just be pretty tired and that you may have some skin irritation. And that’s my understanding.” (Preoperative, Age 65)*

For example, participants understood they would need to undergo radiation every day, but postoperatively they said that radiation-associated fatigue interfered with work and family responsibilities, which they were not as prepared for.

*“I was very surprised I was exhausted…I struggled because I tried to do things. I was trying to walk around a lot and then I found out that I was tired. I wouldn’t push myself. I walked to and from to my appointments, so I always had a little bit of time moving around. That was enough.”(6 weeks postoperative, Age 66)*

Despite pretreatment consultation with their radiation oncologist, participants experienced more psychological and long-term physical effects following the completion of radiation than they had expected.

Most participants received chemotherapy and/or hormone therapy as part of their treatment. Although interviews focused on lumpectomy and radiation, participants did express specific expectations about side effects of chemotherapy and hormone therapy (e.g., menopause and neuropathy).

### Appearance

Some participants described broad or general expectations about the appearance of their breasts (e.g., that they would be fine or look different), whereas others had specific expectations.

*“After surgery, I know the one that I’m having surgery on will be slightly smaller and will look different.” (Preoperative, Age 54)*

With regard to breast appearance after BCT, many participants did not know what to expect and simply answered “I don’t know.” Participants who were more concerned with survival than the appearance of their breasts often expressed indifference to the changes that would result from surgery (4 participants). Some participants expected specific changes, such as scarring, indentations, and change in size, and several described a fear of disfigurement. Postoperatively, some participants were surprised by a satisfying breast appearance.

*“I didn’t know what my breast was going to look like, and I was quite surprised and pleased it looks pretty normal.” (13 months postoperative, Age 59)*

Radiation often had a more negative effect on breast appearance than surgery. Some participants interviewed 1 year after surgery discussed an unexpected decrease in size and permanent discoloration resulting from radiation. One participant expressed interest in the cosmetic options available, owing to dissatisfaction with the unexpected changes she experienced from radiation.

Participants often discussed body image in relation to changes in breast appearance. Several preoperative participants felt that the effect of treatment on their body image was not going to be significant (7 participants). Participants often minimized the effect of surgery on their body or were not concerned with changes. Postoperatively, body image was part of a larger constellation of psychosocial and physical changes. For example, participants expressed that related weight gain, effects of hormone therapy, anxiety, and depression had an effect on how they felt about their body. This directly affected their desire for intimacy and sexual activity.

### Psychosocial and sexual well-being

Participants anticipated changes to relationships during treatment, such as increased support from family and friends and sharing of household responsibilities.

*“I think it will bind us more together...with my daughter.” (Preoperative, Age 60)*

Two participants expected that they would be less likely to see friends and family or that they would be less social. Postoperative participants discussed unexpected changes to their psychosocial well-being. Some of these changes were temporary, but many participants worried about recurrence and noted that cancer permanently altered their life goals. Some participants revealed that treatment had a positive psychological effect (4 participants) bringing them closer to friends and family, whereas others were still recovering emotionally and still learning how to live life after cancer diagnosis and treatment.

When asked about sexual well-being, participants did not have any expectations or didn’t expect change. This topic was not often included in preoperative teaching. However, postoperatively, participants discussed unexpected side effects, such as vaginal dryness, dyspareunia, weight gain or loss, and a general lack of desire for sexual intimacy. Participants had different ways of coping with side effects and the ongoing effects of treatment on their HR-QOL.

## Discussion

This study aimed to explore HR-QOL expectations of patients undergoing BCT for treatment of early stage breast cancer. Preoperatively, patients in our study had a limited understanding of what to expect from BCT. They often prioritize short-term recovery and survival, including concerns about recurrence. Despite preoperative teaching and access to information about lumpectomy and radiation, participants in our study could not easily express their expectations about the effects that surgery and other treatment would have on their body. We found a notable knowledge gap among women undergoing BCT with regard to the long-term impact treatment can have on HR-QOL. The literature suggests that changes to HR-QOL do occur following BCT [5, 10, 11, 22, 32]—the experiences of participants in our study support these findings.

Previous studies have associated BCT with lower psychosocial distress, compared with more-extensive surgical treatments; however, regardless of surgical treatment, distress is high in patients with breast cancer [14, 22, 31]. Furthermore, anxiety and depression is elevated in patients newly diagnosed with breast cancer compared to age-matched groups and remains high in some women 1 year after surgery [8–10, 21, 28]. Our study supports the previous findings of elevated anxiety levels in cancer patients before surgery and may have contributed to the lack of expectations we observed. Patients with high levels of anxiety preoperatively may have difficulty processing information, which may lead to unmet expectations and, as a result, depressed HR-QOL. Patients with anxiety may be at risk for dissatisfaction and clinicians should be aware of this relationshi p[28]..

Participants in our study had access to various sources of information but still had few expectations about long-term HR-QOL. Some participants reported that their expectations were formed using information they received from close friends or family members who had been through breast cancer treatment. Consistent with the literature, participants in our study referenced discussions with their surgeon about surgical decisions and information [16]. Participants interviewed postoperatively relied on information from their radiation oncologist and medical oncologist to form expectations about the impact of radiotherapy, chemotherapy, and hormone therapy on their short and long-term physical and mental health. However, patients often do not meet with members of the medical team beyond their surgeon until after surgery. Making patient-reported outcomes data and information on expected changes to long-term HR-QOL a focus of pretreatment teaching may help patients form expectations beyond recovery from surgery.

Preoperative teaching is focused on short-term recovery and side effects, and this was reflected in patients’ expectations about pain and activities of daily living. Although BCT is often promoted as a less-obtrusive form of treatment, we found that the complete treatment experience, and the recovery from it, can have a great impact on long-term HR-QOL. This was consistent with the findings from a previous study, which showed that patients receiving radiation and/or chemotherapy took, on average, 1 year to complete treatment and that the effects of chemotherapy continued until the end of the study, at 24 months [14]. Side effects reported as unexpected by our participants—such as breast discoloration, fatigue, and the effects of hormone therapy on psychosocial and sexual well-being—are, in fact, well-documented side effects [11, 18, 20, 22]. More preoperative counseling and education about the effects of adjuvant treatment would likely be beneficial to patients undergoing BCT.

This study has limitations. The participants came from a single high-volume institution in the US and had good access to health care in general. Although the sample was diverse in age, race, and ethnicity, all participants spoke English. Thus, our findings have limited generalizability. Another limitation was recall bias among participants interviewed postoperatively. It was difficult for some women to remember what they expected preoperatively; therefore, we focused on unexpected events in the postoperative group.

Our results can be used by clinicians to understand the expectations of BCT patients. Evaluating expectations preoperatively may provide clinicians a way to identify patients who either lack or have misguided expectations about BCT. By identifying these patients preoperatively, clinicians will have the opportunity to intervene with teaching materials that cover long-term HR-QOL outcomes. The use of the participant quotations from this study may help future patients form more-accurate expectations.

## Conclusions

Our study identified a knowledge gap among our participants undergoing BCT for breast cancer that may contribute to adverse HR-QOL outcomes and dissatisfaction. While limited, our results can inform innovative preoperative teaching to help clinicians and patients understand the short- and long-term HR-QOL outcomes following BCT for breast cancer.

## Data Availability

Data sharing is not applicable to this article as no datasets were generated or analyzed during the current study.

## Abbreviations

BCT: Breast-conserving therapy
HR-QOL: Health-related quality of life
